# The Theory and Practice of Consultation-Liaison (CL) Psychiatry in Trinidad and Tobago with Reference to Suicidal Behavior

**DOI:** 10.1100/tsw.2008.74

**Published:** 2008-09-30

**Authors:** Hari D. Maharajh, Petal Abdool, Rehanna Mohammed-Emamdee

**Affiliations:** ^1^ University of West Indies, St. Augustine, Trinidad and Tobago; ^2^ Department of Psychiatry, University of Toronto, Canada; ^3^ Psychiatry Unit, San Fernando General Hospital, Trinidad and Tobago

**Keywords:** consultation-liaison, psychiatry, theory, practice, suicidal behavior, general hospital, Trinidad and Tobago

## Abstract

Consultation-Liaison Psychiatry (CL Psychiatry) is not a well-established discipline in developing countries. It is a multifaceted area that incorporates clinical, teaching, and research activities both within the hospital and extramurally in community health services. Our purpose was first to define the role of CL Psychiatry, to review essential steps in the process, and to advise on how to set up services. Second, a 1-year retrospective analysis was conducted on all patients referred. A total of 708 persons were referred for psychiatric consultation, of which 41% (291) were referred because of suicidal behavior. Sixty-six percent were female and there was an over-representation of Indo-Trinidadians (67%). Twenty-six percent of all cases of suicidal behavior were diagnosed with clinical depression, 3% were suffering from a psychotic illness (schizophrenia), and 8% (24/291) were under the influence of alcohol. The most vulnerable group was the 25- to 35-year-old age group, accounting for 27% (78/291) of attempters, with the largest number of female attempters. The 36- to 55-year-old males were most likely to attempt suicide (35/99). Ingestion of a toxic substance was the most popular method among all races, genders, and age groups. Of all referrals, 95% originated from medical wards. The most common reason cited for attempts was depressed mood, secondary to a domestic dispute with a family member or significant other. Among Caribbean countries, Trinidad and Tobago has high rates of suicide and suicidal behavior, depression, and alcoholism. CL psychiatrists have a major role to play in the delivery of services to these groups, facilitating the transition of care from admission in the emergency room to discharge and follow-up in the community.

## INTRODUCTION

Consultation-Liaison Psychiatry (CL Psychiatry) is not well established in Trinidad and Tobago. Notwithstanding the endorsement of the General Medical Council of the U.K. of liaison psychiatry as a subspecialty within general psychiatry, and the approval of psychosomatic medicine, the preferred terminology, as a subspecialty field of psychiatry by the American Board of Medical Specialties in 2003[1], little attention has been given to this area of practice and research in our setting.

At St. Ann's Hospital in Trinidad, a 1000-bed psychiatric hospital, there is no training or service in CL Psychiatry despite previous attempts to establish a consultation service with the Port of Spain General Hospital[2]. At the San Fernando General and Scarborough Hospitals, there are routine services in CL Psychiatry, but unfortunately, these services are managed by junior doctors with inadequate training, thereby defying the basic principles that consultations are made and done by consultants.

CL Psychiatry is defined as the area of clinical psychiatry that encompasses clinical, teaching, and research activities of psychiatrists and allied mental health professionals in the nonpsychiatric divisions of a general hospital. Over the years, CL services have expanded to provide care in health care facilities other than the general hospital, such as community health clinics, rehabilitation centers, convalescent hospitals, nursing homes, and doctors' private offices. Within hospitals, CL services may be established with a monodisciplinary or medical model, or multidisciplinary or mental health model team[3].

CL Psychiatry started in the U.S. in the 1930s as an outgrowth of general hospital psychiatric units. The first viable general hospital psychiatric unit in the U.S. was opened in the Albany Hospital in 1902. Following that, the Rockefeller Foundation funded the establishment of five psychiatric liaison departments in 1934[4,5].

The development of CL Psychiatry was aided by the emergence in the 1920s of psychosomatic medicine. Before 1950, Franz Alexander's concept of psychosomatic medicine dominated, proposing a *casual link* between a specific constellation of unconscious conflicts, of psychological methods of coping with them, and the development of one of several organic diseases[6]. He proposed that these unconscious conflicts that produce anxiety are mediated through the autonomic nervous system, thereby producing a specific disorder. Between 1951 and 1965, the Chicago Institute of Psychoanalysis postulated a "psychosomatic specificity hypothesis" for a number of physical diseases[6].

The field of CL Psychiatry has grown from a trickle to a flood at the present time. The extension of CL services to the community and other medical facilities, and the de-emphasis on institutionalization has relegated psychiatric units in general hospitals as relics of the past. The role of CL Psychiatry is defined by the American Hospital Association (1970) as follows:

> *The development of liaison psychiatric services is based on the acknowledged fact that psychiatry applied in general or specialty medicine contributes to the quality of care provided, affects hospital utilization, and results in a savings of the physician's time…the liaison psychiatrist often serves the unserved, by helping to ensure the identification and appropriate management of mental and emotional aspects of illness throughout the hospital.*

Today, there is overwhelming evidence to show that psychological factors are major determinants of the presentation and consequences of physical illness and medically unexplained symptoms. There is also a high prevalence of diagnosable psychiatric disorders in in-patient and out-patient populations, which is associated with significant disability and increased use of resources. Hence, the critical role of CL Psychiatry cannot be overemphasized.

The term "Consultation-Liaison" reflects two interrelated roles of the consultant. "Consultation" refers to the provision of expert diagnostic opinion and advice on management regarding a patient's mental state and behavior at the request of another health professional. "Liaison" refers to a linking-up of groups for the purpose of effective collaboration. The role and function of the CL psychiatrist is threefold. It involves clinical work, research, and teaching.

## CLINICAL WORK

CL psychiatrists are involved in the following:

- The diagnosis, prevention, and management of mental illness in physically ill patients. Common CL problems encountered include suicide attempts or threats, depression, agitation, hallucinations, sleep disorders, disorientation (usually secondary to delirium or dementia), or patients presenting with somatic complaints for which there is no organic basis (somatoform disorders).
- The assessment and management of atypical presentations of psychiatric disorders due to medical, neurological, and surgical illnesses and their treatments.
- The assessment of the capacity of patients to give informed consent for medical and surgical procedures.
- To provide nonpharmacological interventions to patients experiencing the emotional effects of their medical conditions.
- To provide mediation between patients and members of the clinical team and to allay conflicts between them.
- To act as mediators and bridge builders between nonpsychiatric and psychiatric professionals.

## RESEARCH

Liaison psychiatrists have made numerous research contributions related to the psychiatric aspects of AIDS; cancer; transplantation; cardiology; neurology; pulmonary, renal, and GI diseases; and obstetrics and gynecology. Their research has extended our knowledge about genetic, neurochemical, and behavioral factors contributing to the development of psychiatric disorders among complex, medically ill patients.

## TEACHING

CL psychiatrists are involved in teaching medical students, physicians, nurses, social workers, psychiatric residents, and nonpsychiatric residents in internal medicine, family practice, pediatrics, obstetrics and gynecology, and neurology.

Crucial skills taught include interviewing techniques, approach to comprehensive diagnosis and management, personality assessment, proper use of drugs, and brief psychotherapy. Included is staff education in psychological aspects of illness. The Psychiatry Unit at Mt. Hope is dedicated to this vision (see Table 1).

## SETTING UP A CL UNIT

A CL service should be an administrative unit treated as an integral component of every psychiatric service, be it a psychiatric unit of a general hospital, a community health clinic, or forensic service. In the multidisciplinary team model, a CL service should be staffed by psychiatrists, liaison nurses, social workers, and psychologists. The number of people involved will depend on the size of the hospital or population served, and the availability of staff and funds. The person in charge should be a senior psychiatrist who is experienced in CL Psychiatry, able to coordinate the activities of the service, and able to negotiate with other physicians on an equal footing. Their duties are full-time liaising with physicians, families, NGOs, community groups, and members of the multidisciplinary team. Consultations are a priority and ought not to be fit in as time out from the consultants' lucrative private practices. On the other hand, physicians should descend from their exalted state and not perceive the psychiatrist to be of less value. The consultant should define which patients have to be seen and what services are to be delivered. The direct and indirect functions of the unit should be defined with predetermined responsibilities for medico-legal issues.

**TABLE 1**
**Essential Steps in the Consultation Process**

Before the interview

- Use structured referral forms
- Review medical records/charts thoroughly
- Talk with referring doctor, nursing staff, or other medical staff
- Identify the reason for referral
- Formulate objective of psychiatric consultation
- Check whether patient is informed about consultation

Interview with the patient

- Introduce yourself or be introduced by referring physician
- Inform patient about the purpose and procedure of consultation
- Assess and consider the patient's anxieties and expectations about the consultation; obtain agreement from patient
- Perform psychiatric assessment
- If appropriate, interview relatives and friends
- Inform the patient about your impressions, diagnoses, and recommended treatment
- Inform the patient about follow-up visits and/or your availability

After the interview

- Make short notes in the medical records
- Inform referring doctor/staff comprehensively about the consultation and your final opinion (diagnosis and formulation)
- Discuss your treatment recommendations thoroughly
- Clarify responsibilities for carrying out your recommendations
- Ensure feasibility of your recommendations
- Make appointment for next follow-up visit
- Write a concise consultation note

Liaison Psychiatry should be funded and managed alongside other medical specialties with equity in funding for all services. Ideally, the psychiatrist should be a formal member of the medical organization of the hospital.

## OBJECTIVE

The purpose of this study is twofold. First, to review the present status of CL Psychiatry locally and regionally with respect to its development as a subspecialty, to define its role, to walk health professionals through the essential steps in the consultation process, and to advise on the establishment of a CL unit. Second, to investigate some sociodemographic factors on consultations of suicidal behavior at a general hospital. Referral for suicidal behavior is the most common cause for consultation in the general hospital and psychiatric clinics in Trinidad and Tobago.

## METHODS

A 1-year (January–December 2005) retrospective analysis was done on all patients referred to the psychiatric unit at the San Fernando General Hospital in the CL service. An analysis was done on patients with suicidal behavior since this constitutes almost half of the patients seen in CL Psychiatry. An analysis was conducted on the method, type of substances used, and demographic data of age, gender, and ethnicity using the SPSS (Statistical Package for the Social Sciences. Version 11.0).

## RESULTS

During the period of January–December 2005, a total of 708 patients were referred for psychiatric consultation. Among this population, 291 (or 41%) were referred because of suicidal behavior. Sixty-six percent (192) or twice as many women attempted suicide than men (34%). Indo-Trinidadians were over-represented in the referrals for suicidal behavior with 195 of 291 (67%) requests for consultation being for people of Indian origin. Indo-Trinidadian males and females outnumbered Africans in all areas.

### Diagnosis

Twenty-six percent (26%) of all cases of suicidal behavior were diagnosed with clinical depression and 3% were thought to be suffering from a psychotic illness (schizophrenia). Eight percent (8%) (24/291) of all suicidal attempts were carried out under the influence of alcohol and these were all older Indo-Trinidadian males from rural areas. Adjustment disorders due to stressors were common among adolescents, but no figures were available.

### Age Categories

Analysis according to age groupings revealed that the most vulnerable group was the 25- to 35-year-old age group, accounting for 27% (78/291) of attempters. This age group also had the largest number of female attempters. The 36- to 55-year-old males were most likely to attempt suicide (35/99). Fifteen percent (15%) of all cases occurred in the <20 years age group, with the expected female predominance. Only 4% of cases were older than 55 years (see Table 2, Fig. 1).

### Method Used

There were only four cases of attempted hanging for the entire year and two cases of self-immolation (burns). Ingestion of a toxic substance was the most popular method among all races, genders, and age groups. The four most popular substances ingested were weedicide/gramoxone 34%, bleach or household cleaner 9%, prescription drugs 52%, and kerosene/diesel oil/battery acid (4%). Weedicide/gramoxone was the most popular method among males, the majority of whom were of East Indian descent. Among the females, ingestion of tablets accounted for 55% of this population, with East Indians again showing the highest prevalence rates (Table 3, Fig. 2).

**TABLE 2**
**Parasuicide for 2005 According to Age Group and Gender**

| | <20 Years | 20–24 Years | 25–35 Years | 36–55 Years | >55 Years |
|---|---|---|---|---|---|
| Male | 13 | 23 | 31 | 35 | 2 |
| Female | 32 | 29 | 47 | 33 | 9 |
| Total | 45 | 52 | 78 | 68 | 11 |

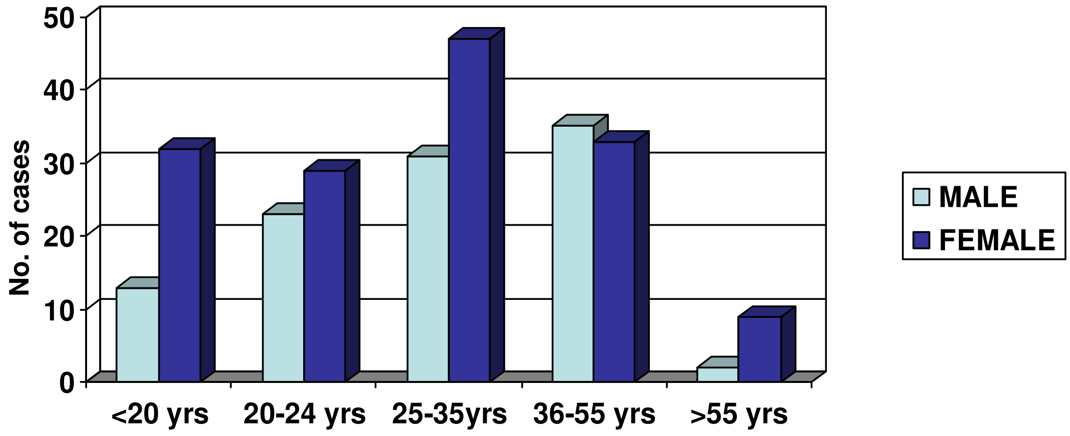

**FIGURE 1.** Bar chart illustrating data from Table 2.

**TABLE 3**
**Toxic Substances Ingested According to Sex and Race, in Percentages***

| | Household Cleaner | Insecticide or Gramoxone | Kerosene or Diesel Oil | Prescription Drugs |
|---|---|---|---|---|
| African male | 4 (1%) | 10 (3.6%) | 2 (0.7%) | 4 (1%) |
| African female | 4 (1%) | 10 (3.6%) | 2 (0.7%) | 20 (7.2%) |
| E. Indian male | 6 (2%) | 40 (14.5%) | 3 (1%) | 28 (10.1%) |
| E. Indian female | 16 (6%) | 30 (10.8%) | 3 (1%) | 94 (34%) |

* Percentages were obtained by dividing by total number of ingestion cases.

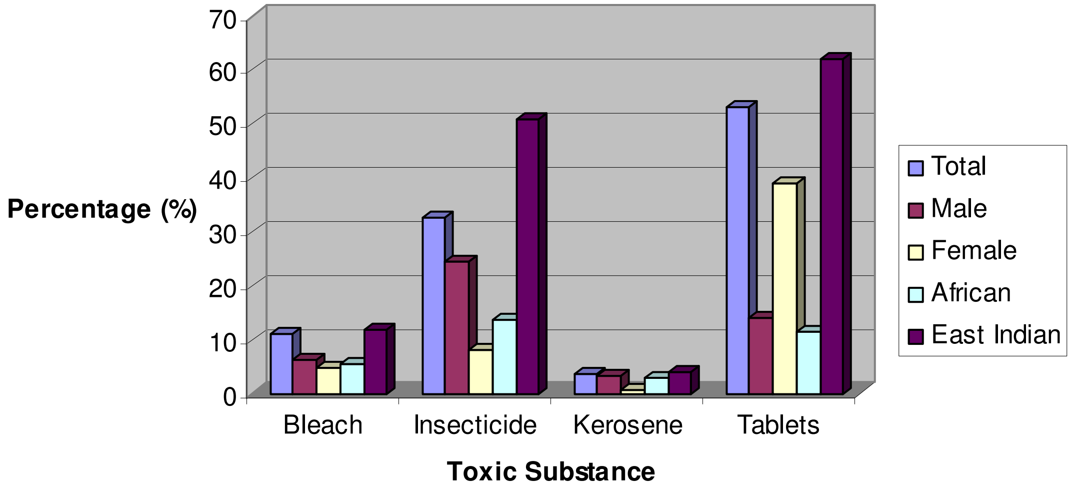

**FIGURE 2.** Bar chart illustrating substances ingested as a percentage of total cases of ingestion.

## Source of Consultations

More than 95% of all referrals originated from the medical wards of this General Hospital for 2005. The most common reason cited for attempted suicide was depressed mood, secondary to a domestic dispute with a family member or significant other.

## DISCUSSION

Trinidad and Tobago are twin islands located in the southern Caribbean Sea just off the coast of Venezuela. Formerly a British colony, the islands attained independence in 1962 and republican status in 1975, and cover a land area of 1,932 $mi^2$. The total population is approximately 1.3 million with 40.3% of the population being of East Indian descent, 39.6% of African origin, 18.4% mixed, and 1.7% belonging to other ethnic groups[13]. The country is classified as a more developed country (MDC), with an economy based principally on oil and gas, and a per capita gross domestic product (GDP) of U.S. $8,948 and total health expenditure of 5.2% of the GDP[7].

Trinidad and Tobago has the second highest rate of suicide in the Caribbean. According to a WHO World Health Report[8], the twin-island republic has a suicide rate of 11.6/100,000 and is only exceeded by Cuba. A more recent study reported the overall mean suicide rate calculated for the period of 1990–1997 to be 20.6/100,000 for the male population and 5.4/100,000 for the female[9]. Organophosphate poisoning with paraquat ingestion is the most common form of self-poisoning. Mortality rates due to self-poisoning by paraquat ingestion accounted for more than 80% of the deaths in South Trinidad[10]. Regional data from Trinidad and Tobago for the years 1986–1990 indicated that paraquat was the most popular poison used in 63% of suicidal attempts and other biocides were used in 20% of the cases[10]. In 1996, 48 cases of suicide were reported with 81.3% due to paraquat poisoning[11].

The Southern and Central regions of Trinidad are less urbanized areas situated in the agricultural belt and comprise approximately half the population of the twin-state republic. The San Fernando General Hospital is the major hospital servicing the southern region and serves a population of approximately 600,000 people with a 60% Indo-Trinidadian residency.

With this background, it is not surprising that of the 708 patients seen for CL Psychiatry at the San Fernando General Hospital in 2005, 41% of the consultations were for suicidal behavior and of the 810 in-patient referrals made in 2006, a similar percentage (43%) was for suicidal behavior. A detailed analysis of 2005 revealed a predominance of females, with 66% females referred for postadmission psychiatric consultation. With respect to the nature of poisonous substances used, of those seen in CL Psychiatry on the wards, 34% used pesticides, 52% oral medication, 9% cleaning agents, and 5% kerosene. Approximately 8% of the CL population carried out their acts under the influence of alcohol. These were mostly older males of East Indian origin.

In Trinidad and Tobago, suicidal behavior is a common finding among the Indo-Trinidadian population. More than 95% of all referrals originated from the medical wards of the General Hospital. Sixty-seven percent (67%) of all requests were for people of Indian origin. The mean age of all attempters was 23.4 years, underlining a need to address the problems of substance abuse and poisoning among adolescents in structured community CL services.

Despite the global burden of pesticide poisoning and a proactive initiative by the WHO[12], little attention has been given to pesticide poisoning in Trinidad and Tobago. This is in part due to lack of government investment in the agroindustries and its pursuit of policies of political expediency rather than human suffering. The widespread use and availability of pesticides with poor restriction and control has resulted in high suicide rates in developing countries[13]. This is evident in Trinidad and Tobago. This further underlines the need to invest in CL community psychiatry.

The European Consultation-Liaison Workshop for General Hospital Psychiatry and Psychosomatics[14] has identified two groups of patients, namely attempted suicide and substance abuse patients, that make up between a quarter to a third of those referred for CL service. In this study, 41%

were referred for attempted suicide, of which 8% were under the influence of alcohol and 26% were found to be depressed. Since these disorders are common in Trinidad and Tobago[15,16], CL psychiatrists have a major role to play in the delivery of service to these groups, facilitating the transition of care from admission in the emergency room to discharge and follow-up in the community.

There is a changing culture within general hospitals both in the developing and developed world. There is a change in functions of the hospital with a shift to a client-oriented service, new technologies requiring specialized skills, implementation of organizational structures, and the need to put effective economic measures into place. This changing trend on a background of the closure or downsizing of large mental institutions has placed greater demands on the general hospitals[17]. There is now an influx of more patients for treatment, reduced length of stay in the hospital, an increase in the proportion of severely disordered patients, and, consequently, an increase in suicidal behavior within the hospital setting. While education and extensive screening programs are mandatory at all levels in the community, the time has come for the establishment of a CL rolling program for the early and vigorous treatment of patients both at the community and hospital levels.

Given the importance of CL Psychiatry and the vast advances that have been made in this field in recent times, it is regrettable that formal training in this area is lacking and/or inadequate in Trinidad and Tobago. This matter is currently under review at the University of the West Indies Psychiatry Unit at Mt. Hope in Trinidad, and we hope this publication and others[18,19,20,21,22,23,24,25,26,27,28,29,30,31] in this journal will be helpful in the decision-making process.

## ACKNOWLEDGMENTS

We wish to thank Dr. Shivanand Gopeesingh, consultant in ER trauma at the San Fernando General Hospital for the provision of data for this study, and the Regional Health Authority for permission to publish this study.

## References

1. Gitlin, D.F., Schindler, B.A., et al. (2004) Psychosomatic medicine: a new psychiatric subspecialty. *Acad. Psychiatry* **28,** 4–11.
2. Maharajh, H.D. (1999) A Proposal for Registrars and Specialist Medical Officers to be on Call at the Port of Spain General Hospital Following Closure of Ward 8. Policy statement submitted to the NWRHA from the Medical Chief of Staff Office. Archives. St. Ann's Hospital, Trinidad.
3. Huyse, F.J. (2000) *The Organization of Psychiatric Services for General Hospital Departments.* Vol. 2. Gelder, M.G., Lopez-Ibor, J.J., and Andreasen, N., Eds. Oxford University Press. pp. 1237–1242.
4. Lipowski, Z.J. (1974) Consultation-liaison psychiatry: an overview. *Am. J. Psychiatry* **131,** 623–629.
5. Lloyd, G. and Mayou, R. (2003) Liaison psychiatry or psychological medicine? *Br. J. Psychiatry* **183**, 5–7.
6. Buckley, P., Bird, J., and Harrison, G. (1996) *Examination Notes in Psychiatry – A Post Graduate Text.* 3rd ed. Butterworth-Heinemann. pp. 222–233.
7. World Health Organization (2002) Project Atlas: Country Profile-Trinidad and Tobago. WHO, Geneva. Available at www.cvdinfobase.ca/mh-atlas/.
8. World Health Organization (2001) World Health Report. October 2001. WHO, Geneva.
9. Ameerali, D. (2002) Suicide in Trinidad and Tobago: An Ecological Study [Doctor of Medicine Thesis]. Faculty of Medical Sciences, University of the West Indies, St. Augustine, Trinidad.
10. Hutchinson, G., Daisley, H., Simmons, V., and Gordon, A.N. (1991) Suicide by poisoning. *West Indian Med. J.* **40(2),** 69–73.
11. Hutchinson, G., Daisley, H., Simeon, D., Simmonds, V., Shetty, M., and Lynn, D. (1999) High rates of paraquat-induced suicide in southern Trinidad. *Suicide Life Threat. Behav.* **29(2),** 186–191.
12. World Health Organization (2004) The Prevention of Mental Disorders: Effective Interventions and Policy Options. WHO, Geneva.
13. Bertolote, J.M., Fleischmann, A., Eddleston, M., and Gunnell, D. (2006) Deaths from pesticide poisoning : a global response. *Br. J. Psychiatry* **189,** 201–203.
14. Bassett, D. and Tsourtos, G. (1993) Inpatient suicide in a general hospital psychiatric unit: a consequence of inadequate resources? *Gen. Hosp. Psychiatry* **15(5),** 301–306.

15. Maharajh, H.D. and Ali, A. (2006) Adolescent depression in Trinidad and Tobago. *Eur. Child Adolesc. Psychiatry* **15**, 30–37
16. Maharajh, H.D. and Konings, M. (2005) Cannabis and suicidal behaviour among adolescents. In *Suicidal Behaviour in Adolescence: An International Perspective*. Merrick, J. and Zalsman, G., Eds. Freund Publishing, London.
17. Murray, C.J.L. and Lopez, A.D. (1996) The global burden of disease - a comprehensive assessment of morality and disability from diseases, injuries and risk factors in 1990 and projected to 2020. In *Global Burden of Disease and Injury.* Series. Vol. 1. Harvard School of Public Health, Cambridge, MA.
18. Ventegodt, S. and Merrick, J. (2005) Suicide from a holistic point of view. *TheScientificWorldJOURNAL* **5,** 958–966.
19. Merrick, J. and Zalsman, G. (2005) Suicide from an international perspective. *TheScientificWorldJOURNAL* **5,** 888–890.
20. Latzer, Y. and Hochdorf, Z. (2005) A review of suicidal behavior in anorexia nervosa. *TheScientificWorldJOURNAL* **5,** 820–827.
21. Dancyger, I.F. and Fornari, V.M. (2005) A review of eating disorders and suicide risk in adolescence. *TheScientificWorldJOURNAL* **5,** 803–811.
22. Maharajh, H.D. and Abdool, P.S. (2005) Cultural aspects of suicide. TheScientificWorldJOURNAL **5,** 736–746.
23. Merrick, J., Merrick, E., Lunsky, Y., and Kandel, I. (2005) Suicide behavior in persons with intellectual disability. *TheScientificWorldJOURNAL* **5,** 729–735.
24. Merrick, J., Merrick, E., Morad, M., and Kandel, I. (2005) Adolescents with intellectual disability and suicidal behavior. *TheScientificWorldJOURNAL* **5,** 724–728.
25. Shek, D.T.L., Lee, B.M., and Chow, J. (2005) Trends in adolescent suicide in Hong Kong for the period 1980 to 2003. *TheScientificWorldJOURNAL* **5,** 702–723.
26. Shahtahmasebi, S. (2003) Homicides and suicides by mentally ill people. *TheScientificWorldJOURNAL* **3,** 684–693.
27. Morad, M., Merrick, E., Schwarz, A., and Merrick, J. (2005) A review of suicide behavior among Arab adolescents. *TheScientificWorldJOURNAL* **5,** 674–679.
28. Shahtahmasebi, S. (2005) Suicide in New Zealand. *TheScientificWorldJOURNAL* **5,** 527–534.
29. Birnbaum, L. (2005) Adolescent aggression and differentiation of self: guided mindfulness meditation in the service of individuation. *TheScientificWorldJOURNAL* **5,** 478–489.
30. Pompili, M., Girardi, P., Lester, D., and Tatarelli, R. (2006) Suicide soon after generalized tonic-clonic seizure. A possible peri-ictal phenomenon? *TheScientificWorldJOURNAL* **6,** 356–360.
31. Birnbaum, L. and Birnbaum, A. (2004) In search of inner wisdom: guided mindfulness meditation in the context of suicide. *TheScientificWorldJOURNAL* **4,** 216–227.
